# Adherence to a Vegetarian Diet and Diabetes Risk: A Systematic Review and Meta-Analysis of Observational Studies

**DOI:** 10.3390/nu9060603

**Published:** 2017-06-14

**Authors:** Yujin Lee, Kyong Park

**Affiliations:** Department of Food and Nutrition, Yeungnam University, Gyeongsan 38541, Gyeongbuk, Korea; yj_lee@yu.ac.kr

**Keywords:** vegetarian, diabetes, systematic review, meta-analysis

## Abstract

We quantitatively assessed the association between a vegetarian diet and diabetes risk using pooled estimates from observational studies. Electronic database searches for articles published from January 1980 to May 2016 were independently performed by two investigators, and 13 articles (14 studies) were identified. The pooled odds ratio (OR) for diabetes in vegetarians vs. non-vegetarians was 0.726 (95% confidence interval (CI): 0.608, 0.867). In the subgroup analyses, this inverse association was stronger for the studies conducted in the Western Pacific region (OR 0.514, 95% CI: 0.304, 0.871) and Europe/North America (OR 0.756, 95% CI: 0.589, 0.971) than studies conducted in Southeast Asia (OR 0.888, 95% CI: 0.718, 1.099). No study had a substantial effect on the pooled effect size in the influence analysis, and the Egger’s (*p* = 0.465) and Begg’s tests (*p* = 0.584) revealed no publication bias. This meta-analysis indicates that a vegetarian diet is inversely associated with diabetes risk. Our results support the need for further investigations into the effects of the motivations for vegetarianism, the duration of the adherence to a vegetarian diet, and type of vegetarian on diabetes risk.

## 1. Introduction

Diabetes mellitus is one of the largest global disease burdens of the 21st century. According to a report of the International Diabetes Federation in 2015 [1], 415 million adults (aged 20–79 years) were diagnosed with diabetes, and 318 million had impaired glucose tolerance. Furthermore, the global estimate of the number of patients with diabetes is expected to increase to 642 million by 2040. Diabetes is also a major risk factor for cardiovascular disease, one of the leading causes of mortality [2].

Many studies have shown that diabetes can be prevented with a well-balanced and healthy diet and lifestyle [2,3,4], in particular, by adhering to a well-planned vegetarian diet [5]. A vegetarian diet is mostly based on plant foods such as cereals, legumes, fruits, leafy vegetables, nuts, seeds, and sea vegetables. The definition of a vegetarian diet varies from study to study [6,7,8,9,10,11], and vegetarian diets are classified based on patterns of eliminations of food groups such as fish, eggs, and/or dairy products from the diet (pesco-, ovo-, lacto-, and lacto-ovo-vegetarians, respectively). In this meta-analysis, a vegan is defined as someone who consumes no food from animal sources or only does so up to once per month. Dairy products, eggs, both dairy products and eggs, and fish form the parts of lacto-, ovo-, lacto-ovo- and pesco-vegetarian diets, respectively. Semi-vegetarians are defined as individuals who eat meat up to once per week. The most common type of vegetarian differs by country and continent, according to culinary tradition. For instance, most European and North American vegetarians are lacto-ovo-vegetarians [12,13], whereas Asian Indian vegetarians are predominantly lacto-vegetarians [6,14]. Moreover, it has been shown that Chinese vegetarians consume significantly fewer dairy products than Western vegetarians [9,15].

Although the health benefits of vegetarian diets have been well documented, most evidence is from short-term randomized controlled trials (RCT). Previous meta-analysis and reviews on the effects of vegetarian diets on diabetes risk have mainly focused on intervention studies with relative short-term effects [16,17,18]. Although RCTs are referred to as the ‘gold standard’ of evidence-based studies, results from observational studies may be more helpful in understanding the health benefits of a vegetarian diet as they can include data on individuals with long-term adherence and compliance to/with such a diet.

Thus, the aim of this study was to review the available literature and assess the association between a vegetarian diet and the risk of diabetes via a meta-analysis of observational studies. We hypothesized that adherence to a vegetarian diet has an inverse association with diabetes risk.

## 2. Materials and Methods

A systematic review and meta-analysis on the association between a vegetarian diet and the risk for diabetes was performed according to Guidelines for Meta-analyses and Systematic Reviews of Observational Studies (MOOSE). An electronic database search of PubMed, Web of Science, Cochrane Library, and ScienceDirect for literature published between January 1980 and May 2016 was conducted by the two investigators (Y.L., K.P.) independently. The following keywords were used for the searches: “veganism” OR “vegetarianism” OR “vegan” OR “vegetarian” OR “vegetarian diet” OR “vegan diet” OR “plant-based diet” OR “meatless diet” OR “lacto-vegetarian” OR “lactovegetarian” OR “ovo-vegetarian” OR “ovovegetarian” OR “lacto-ovo-vegetarian” OR “lactoovovegetarian” OR “pesco-vegetarian” OR “pescovegetarian” OR “semi-vegetarian” OR “semivegetarian” AND “diabetes” OR “diabetes mellitus” OR “diabetic” OR “type 2 diabetes” OR “type 2 diabetes mellitus” OR “NIDDM” OR “non-insulin dependent diabetes mellitus” OR “noninsulin dependent diabetes mellitus” in all fields and as MeSH terms. Information on all references obtained from the searches was imported into the reference management software EndNote X7 (Thomson Reuters, San Francisco, CA, USA), in which the imported data were organized and selected for meta-analysis via the removal of duplicates and screening by title and abstract. We searched the literature without language restrictions. In addition, we contacted the authors to obtain data that were necessary for our analysis if it was not included in the original article. We screened titles and abstracts and selected studies by reviewing the full texts. Two investigators performed the selection process independently, and differences in opinion between the investigators regarding study selection were resolved by discussion. Manual searches using the reference lists of screened articles were also performed; however, no additional articles were found to be eligible for inclusion in the analysis.

Inclusion criteria for this meta-analysis were as follows: (1) original research; (2) epidemiological study conducted in humans; (3) exposure: vegetarian diet; (4) outcome: prevalence or incidence of diabetes (diabetes was defined as self-reported or physician-diagnosed fasting blood glucose levels ≥ 126 mg/dL (7.0 mmol) or HbA1c levels ≥ 6.5% [19]); and (5) provision of odds ratio (OR), relative risk (RR), or hazard ratio (HR) with 95% confidence intervals (CI).

The following information was extracted from the selected studies (Supplementary Table S1): name of the first author, year of publication, study design, sample size, characteristics of participants or study name, and the country in which the study was performed. Other information included the mean age or age range of the participants, methods of exposure assessment, exposure classification, outcome, assessment method of the outcome, effect size (OR, RR, HR) with 95% CI, and adjusted variables.

The ORs, RRs, and HRs with their 95% CI were used as measures of the association between a vegetarian diet and the risk of diabetes; they were combined for the meta-analysis and presented in a forest plot. Before pooling the risk estimates, heterogeneity was tested based on the Cochran’s Q test (Chi-square analysis) and Higgins I-squared statistics. The Cochran‘s Q test is one of the most frequently used tests of heterogeneity. However, it often has low power to detect true heterogeneity, especially in meta-analyses with a small number of studies. To improve this shortcoming, the significance level was set at *p* < 0.10 instead of *p* < 0.05 [20]. Moreover, the I-square was calculated to quantify the degree of heterogeneity among the studies [21]. In this meta-analysis, if the *p* value of the Cochran’s Q test was < 0.10 or the I-squared statistic > 60%, we considered that heterogeneity existed among studies, and a random effects model was applied [21]. In addition, to reduce heterogeneity and identify the influencing factors of heterogeneity among the study results, subgroup analyses were performed on a subset of participants or studies based on sex, study design, region of study, and type of vegetarian diet. Furthermore, a sensitivity analysis using influence analysis was performed to assess the impact of a single study on the overall pooled estimates by removing one study at a time. To identify a potential publication bias, both visually and statistically, funnel plot asymmetry, Egger’s, Begg’s, and Mazumdar rank correlation tests were conducted.

All data analyses and graph presentations were performed with STATA, version 14 (StataCorp LP, College Station, TX, USA). Unless otherwise stated, *p* < 0.05 was considered statistically significant.

## 3. Results

**Figure 1:**
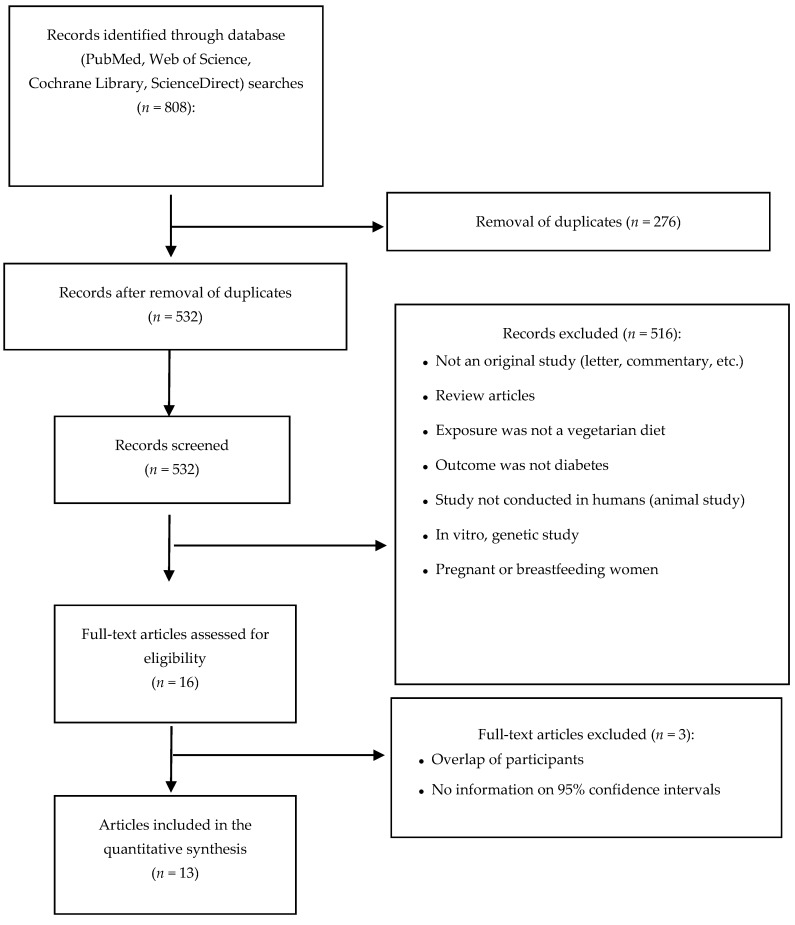
Flow diagram of study selection.

Database searches retrieved 808 articles from 4 electronic databases (PubMed, Web of Science, Cochrane Library, and ScienceDirect). A total of 13 articles including 14 studies (1 article compared the results of 2 studies) met the inclusion criteria [11]. The selection process is presented as a flow diagram, according to the Preferred Reporting Items for Systematic Reviews and Meta-Analysis (PRISMA) (Figure 1).

The main characteristics of the selected studies are summarized in Supplementary Table S1. Of the 14 studies included in our analysis, with 2 cohort [22,23] and 12 cross-sectional studies [6,8,9,10,11,13,24,25,26,27,28]. The results of eight studies indicated that vegetarians had a lower prevalence and incidence of diabetes than omnivores, while no significant associations between a vegetarian diet and diabetes risk were observed in five studies.

**Figure 2:**
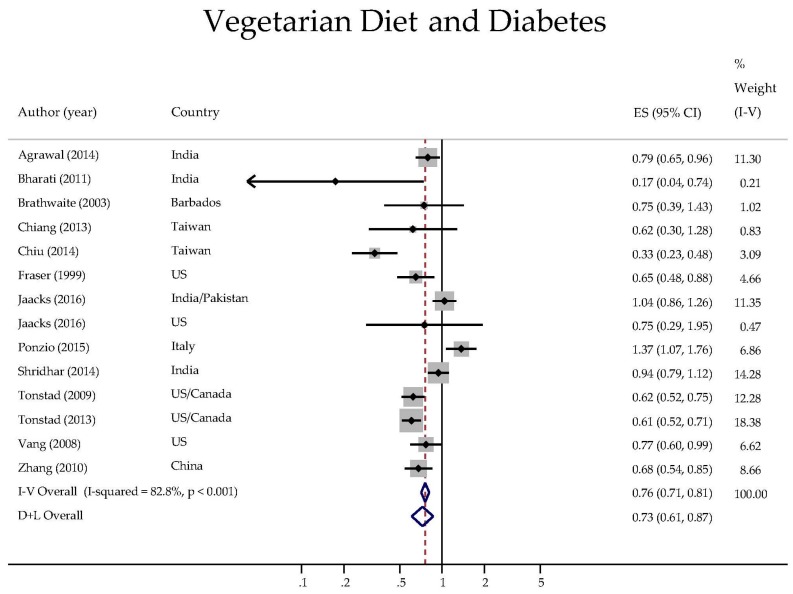
Forest plot of the pooled odds ratios of the association between a vegetarian diet and the prevalence or incidence of diabetes.

Due to the high heterogeneity among the studies, we employed a random effects model to pool the effect estimates (Figure 2). Accordingly, vegetarians had a 27% lower odds of having diabetes than omnivores (OR 0.73, 95% CI: 0.61, 0.87).

In the subgroup analyses by sex, study design, region of study, and vegetarian type, the pooled OR of all subtotal estimates suggested that vegetarians had a lower prevalence or incidence of diabetes than omnivores (Table 1). Vegetarian men were less likely to have diabetes than their omnivorous counterparts; in contrast, we observed no such significant association in women. In the subgroup analysis, the inverse association between a vegetarian diet and diabetes incidence/prevalence tended to be stronger for the three studies conducted in the Western Pacific region (OR 0.514, 95% CI: 0.304, 0.871) and the seven studies performed in Europe & North America (OR 0.756, 95% CI: 0.589, 0.971) than for the four studies conducted in Southeast Asia (OR 0.888, 95% CI: 0.718, 1.099). In the subgroup analysis by types of vegetarianism, most types were significantly associated with a lower prevalence or incidence of diabetes than omnivorous participants, except for pesco-vegetarians.

**Figure 3:**
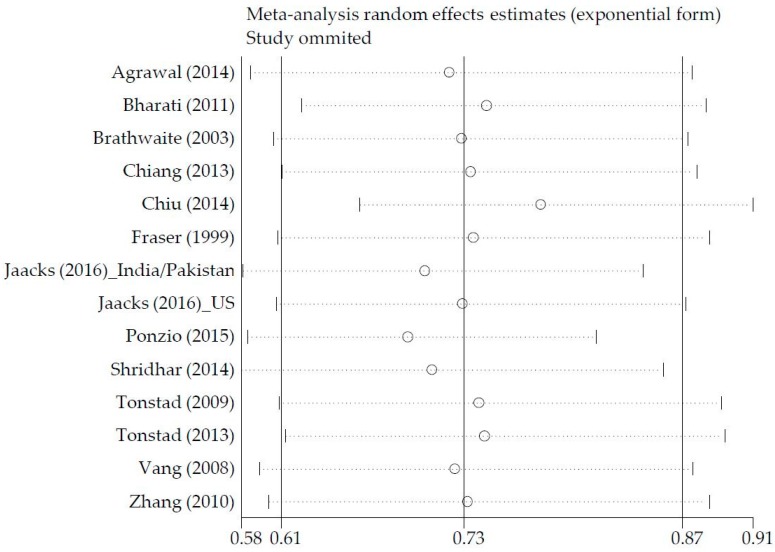
Influence analysis of pooled odds ratios.

The influence analysis showed that the pooled OR was not dramatically changed when it was recalculated after dropping one study at a time (Figure 3). In other words, no one study had a substantial impact on the pooled effect size; this is indicative of a statistically robust result.

**Figure 4:**
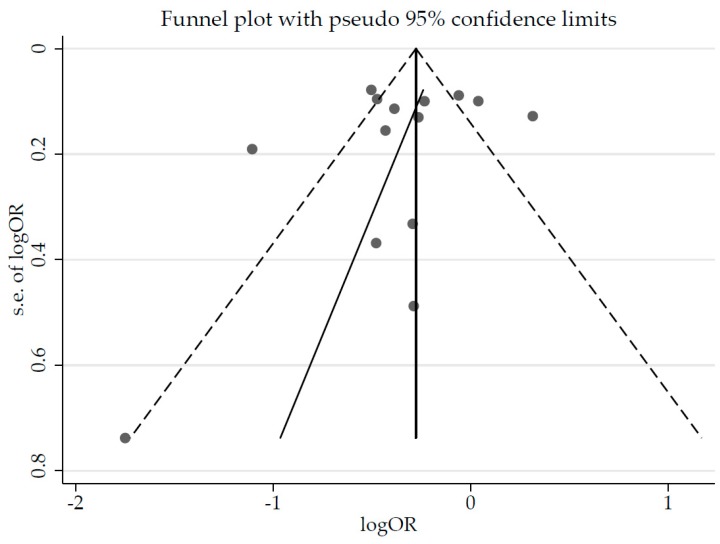
Funnel plot of the estimated publication bias of all studies.

Although the funnel plot showed a slight asymmetry (Figure 4), publication bias was not detected based on statistical tests such as the Egger’s (*p* = 0.465) and Begg’s tests (*p* = 0.584).

## 4. Discussion

This meta-analysis investigated the association between a vegetarian diet and the risk of diabetes based on 2 cohort [22,23] and 12 cross-sectional studies [6,8,9,10,11,13,24,25,26,27,28]. The pooled and subtotal ORs indicated that a vegetarian diet was inversely associated with diabetes incidence/prevalence (OR 0.73, 95% CI: 0.61, 0.87).

Cumulative evidence has emphasized that a vegetarian diet has beneficial effects on diabetes prevention, partly due to the lower body mass index (BMI) of vegetarians when compared to their omnivorous counterparts [8,10,23,24,25]. However, the observed inverse association between a vegetarian diet and the risk of diabetes remained statistically significant after adjusting for BMI [13,22,23]. Other factors of the vegetarian diet might contribute to diabetes prevention and improved insulin sensitivity. For instance, insulin sensitivity was found to be higher in vegetarians than in omnivores; this was negatively associated with the duration of adherence to a vegetarian diet [8,29,30]. Vegans in particular often had the lowest odds of diabetes when compared to other types of vegetarians [13]; they also had lower levels of intramyocellular lipids, which may be related to insulin resistance [31]. Similarly, a meta-analysis of results from intervention studies showed that a vegetarian diet significantly improved HbA1c level in patients with diabetes [16].

Several meta-analyses suggested that whole grains, fruits, and vegetables (in particular, root vegetables and leafy greens, which are high in dietary fibers, beta-carotene, vitamin C, and magnesium) had beneficial effects on diabetes prevention [32,33,34,35]. The most recent meta-analysis of prospective studies using a dose-response analysis revealed that the risk of type 2 diabetes mellitus was reduced by up to 81% by optimally consuming risk-reducing foods such as whole grains, vegetables, fruits, and dairy while simultaneously avoiding risk-increasing foods such as red and processed meats, sugar-sweetened beverages, and eggs [3]. Furthermore, no consumption of risk-increasing foods had more protective effects on developing diabetes than the consumption of risk-reducing foods.

Similarly, other meta-analyses indicated that the positive association between a diet high in red or processed meats and diabetes risk remained after adjusting for the intake of saturated and total fat as well as BMI [23,33,36,37]. It was assumed that nitrosamine and advanced glycation end-products in processed meats might further contribute to the development of diabetes [37,38]. Moreover, meat supplies substantial amounts of heme iron, which accelerates oxidative stress, consequently affecting insulin resistance and glucose metabolism [37].

In the subgroup analysis by vegetarian type, vegan, lacto- and lacto-ovo-vegetarians had a lower risk of diabetes when compared to the omnivore group; however, pesco-vegetarians were not associated with a decreased diabetes risk. One of reasons for this observation could be the heterogeneity of the cooking methods or the type of fish/seafood consumed [22], or a lack of efficacy of this particular type of vegetarian diet. Interestingly, recent meta-analysis showed that the consumption of fish/seafood and type 2 diabetes mellitus risk were negatively correlated in Asians who ate more fatty fish (raw or steamed) but positively correlated in North Americans and Europeans who ate more deep-fried white fish [39].

To understand the pooling estimates regarding the association between a vegetarian diet and the risk of chronic health conditions, some important considerations should be made. First, various types of vegetarians have been included in earlier studies, and inconsistent definitions of the term ‘vegetarian’ have been used. Some authors defined vegetarians as those who never eat meat, whereas others included those who eat meat up to once per week or month. The most common type of vegetarians also varies by geographic and cultural background. For instance, most Indian vegetarians are lacto-vegetarians, while many vegetarians in the US are lacto-ovo-vegetarians [6,11,13,27,40]. Moreover, self-reported information on vegetarianism using dietary questionnaires should be carefully interpreted, because it is often inconsistent with real intake [25,41,42]. For instance, some people defined themselves as vegetarians when they had a vegetarian meal only once daily or even as monthly special events [8]. Third, the quality of the vegetarian diet matters for its health effects [43,44]. Most vegetarians compensate for nutrient deficiencies caused by the removal of food from animal sources with a well-designed vegetarian diet rich in whole grain, legumes, nuts, vegetables, and fruits. However, there are some non-meat-eaters who eat less meat due to economic reasons or other non-health related reasons, who might also be classified as vegetarians [45]. Consequently, the expected positive effects of a vegetarian diet do not always occur. For instance, vegetarians in the US are typically more health-conscious than those in South Asian who traditionally consume food products with a high dairy content (containing high in sugars and fats) [11,46]. Furthermore, some people follow a vegetarian diet due to existing disease. As a result, the association between a vegetarian diet and the disease can be weakened or even show an opposite directional trend [26]. Fourth, the duration of adherence to a vegetarian diet should also be considered. The beneficial health effects of a vegetarian diet on chronic diseases are often related to the duration of adherence to the diet; e.g., long-term vegetarian diet had lower chronic disease or obesity rates than comparable omnivores [25,47]. Information on the length of time of maintaining a vegetarian diet is also particularly useful for supporting the findings from cross-sectional studies.

This study has several strengths. First, to the best of our knowledge, this is the first meta-analysis of observational studies on the association between a vegetarian diet and the risk of diabetes, reflecting relatively long-term exposure stratified by vegetarian type and region. In fact, non-compliance with the intervention is an important limitation of RCTs [48]. Second, multiple databases were used for the literature search, and the two investigators performed the searches independently according to inclusion criteria. Third, all publications were screened by keyword without language restriction, and there was no publication bias.

Our study also had some limitations. First, although heterogeneity among studies typically occurs in meta-analysis, a degree of heterogeneity remained in the subgroup analysis in which varying study results that are likely due to diverse dietary cultures and various definitions of a vegetarian diet were pooled. Second, most studies included in this meta-analysis had a cross-sectional design; thus, a cause–effect relationship could not be assessed. However, the summary estimates of the cross-sectional studies were similar to those from prospective cohort studies, showing that vegetarian diet was protective against diabetes risk. Last, self-reported diabetes was not verified by laboratory testing or clinical diagnosis in all studies, and we did not distinguish between type 1 and type 2 diabetes mellitus.

## 5. Conclusions

The findings of this meta-analysis of observational studies raise the possibility that a vegetarian diet has a protective effect against diabetes risk. However, well-designed prospective cohort studies from various countries that obtain information on the participants’ motivations for vegetarianism, the duration of adherence to a vegetarian diet, and verification of a vegetarian diet are needed to strengthen these findings.

## Figures and Tables

**Table 1:** Pooled odds ratios and 95% confidence intervals for the assocation between a vegetarian diet and diabetes risk by subgroups.

Study	No. of Studies	Odds Ratio (95% CI)		Heterogeneity	
				*p*	*I*^2^ (%)
Sex					
Men	3	0.614	(0.527, 0.716)	0.719	0.0
Women	4	0.569	(0.298, 1.086)	<0.001	86.0
Overall	7	0.584	(0.439, 0.778)	0.001	73.6
Study design					
Prospective cohort	2	0.644	(0.565, 0.735)	0.116	59.5
Cross-sectional	12	0.733	(0.595, 0.904)	<0.001	83.1
Overall	14	0.726	(0.608, 0.867)	<0.001	82.8
Region of study					
Southeast Asia	4	0.888	(0.718, 1.099)	0.03	66.4
Western Pacific	3	0.514	(0.304, 0.871)	0.005	81.1
Europe & North America	7	0.756	(0.589, 0.971)	<0.001	81.9
Overall	14	0.726	(0.608, 0.867)	<0.001	82.8
Vegetarian type					
Vegan	4	0.593	(0.386, 0.911)	0.017	70.5
Lacto-vegetarian	3	0.762	(0.613, 0.949)	0.016	75.7
Lacto-ovo-vegetarian	4	0.564	(0.517, 0.616)	0.301	17.9
Pesco-vegetarian	4	0.867	(0.636, 1.180)	0.008	74.9
Semi-vegetarian	9	0.799	(0.667, 0.956)	0.002	67.3
Overall	24	0.735	(0.654, 0.826)	<0.001	78.8

## Supplementary Materials

The following are available online at www.mdpi.com/2072-6643/9/6/603/s1 , Table S1: Characteristics of studies included in the meta-analysis.
