# Supplementary material for: Adherence to a Vegetarian Diet and Diabetes Risk: A Systematic Review and Meta-Analysis of Observational Studies
Source: Nutrients. 2017 Jun 14;9(6):603. doi: 10.3390/nu9060603 (PMC5490582; doi:10.3390/nu9060603)
Supplement: Supplementary file 1 [file nutrients-09-00603-s001.zip › nutrients-199126-supplementary.pdf]

Supplementary Table S1. Characteristics of studies included in the meta-analysis.

No	First author (publication year)	Study design	Sample size	Participant/ Study name	Country/ Race	Age	Sex	Definition of exposure	Assessment of exposure	Outcome	Assessment of outcome	Odds ratio (95%CI)	Adjusted variables
1	Agrawal (2014)	Cross-sectional	156,317 (M 56,742 W 56,742)	India's 3rd National Family Health Survey (NFHS-3, 2005-06)	India	20-49y	M&W	1) Vegan: never consuming animal products<br>2) LV: consuming fruits, vegetables, pulses or beans, milk or curd, either daily, weekly or occasionally, but no fish, eggs or chicken or meat<br>3) LOV: consuming LV diet and egg<br>4) PV: consuming LOV diet and fish<br>5) SV: consuming fruits, vegetables, pulses or beans, animal products either daily, weekly or occasionally, but no fish<br>6) NV: consuming fruits, vegetables pulses or beans, animal products either daily, weekly or occasionally	Questionnaire on frequency of consumption of selected food	DM	Self-report (not verified)	Men:<br>1) Vegan vs. NV[ref]: 0.70 (0.25, 1.96)<br>2) LV vs. NV[ref]: 0.66 (0.52, 0.82)<br>3) LOV vs. NV[ref]: 0.63 (0.39, 1.00)<br>4) PV vs. NV[ref]: 0.80 (0.43, 1.50)<br>5) SV vs. NV[ref]: 0.45 (0.29, 0.71)<br><br>Women:<br>1) Vegan vs. NV[ref]: 1.01 (0.65, 1.56)<br>2) LV vs. NV[ref]: 0.70 (0.59, 0.82)<br>3) LOV vs. NV[ref]: 0.77 (0.50-1.19)<br>4) PV vs. NV[ref]: 1.33 (0.95, 1.87)<br>3) SV vs. NV[ref]: 1.09 (0.81, 1.47)	Age, gender, education, household wealth, rural/urban residence, religion, caste, smoking, alcohol use, television watching, BMI
2	Bharati (2011)	Cross-sectional	812	Adults not taking any type of anti-diabetic drugs	India	≥30y	M&W	Not clearly defined	Interview (type of food, dietary habit)	DM	1) Fasting glucose level ≥126mg/dl and<br>2) not on treatment	NV vs. V[ref]: 5.738 (1.350, 24.396)	Age, residence, education, tobacco addiction, BMI, waist hip ratio, total blood cholesterol
3	Brathwaite (2003)	Cross-sectional	407	Barbadian Seventh-Day-Adventists	Barbados	25-74y	M&W	1) Self-reported vegetarian(SRV)<br>2) Vegetarian by definition(VBD): consuming meat or poultry <1 d/wk	Self-report	DM	1) Self-report according to physician diagnosis or<br>2) Fasting blood glucose(≥7.8 mmol/L, 140mg/dL)	1) Non-SRV vs. SRV[ref]: 1.27 (0.77, 2.10)<br><br>2) Non-VBD vs. VBD[ref]: 1.34 (0.70, 2.57)	Not available

4	Chiang<br>(2013)	Case-control	706	Age-matched volunteer helpers of Buddhist Tzu Chi<br>Foundation	Taiwan	Mean 56.4y	W	V: practiced vegetarian diet at all meals daily and ≥1y<br>(V+LV+OV+LOV)	Dietary questionnaire	DM	1) Receiving medications for diabetes or<br>2) Fasting glucose levels ≥126 mg/dL	V vs. NV[ref]:<br>0.62 (0.30, 1.27)	BMI, waist, glucose levels, systolic blood pressure, TG, LDL-C, HDL-C, HOMA-IR
5	Chiu<br>(2014)	Cross-sectional	4,384	Taiwanese Buddhist volunteers who receive a free health examination	Taiwan	Mean<br>1) M: V 55y, Omni 55y<br>2) Pre-menopausal W: V 46y, Omni 45y,<br>3) Post-menopausal W: V 59y, Omni 58y	M&W	V: containing no fish, no meat	FFQ	DM	Self-report +<br>1) Physician diagnosis or<br>2) Prescription of diabetes medication or<br>3) Fasting plasma glucose ≥7.0 mmol/L or<br>4) HbA1C≥6.5%	V vs. NV[ref]:<br>1) M:<br>0.49 (0.28, 0.89)<br><br>2) Pre-menopausal W:<br>0.26 (0.06, 1.21)<br><br>3) Post-menopausal W:<br>0.25(0.15, 0.42)	Age, family history of<br>diabetes, education, LTPA, BMI, smoking (men only), alcohol (men only)
6	Fraser<br>(1999)	Cross-sectional	34,192<br>(M 13,857<br>W 20,341)	California Seventh-day Adventists (The Adventist Health Study-1, AHS-1)	US/Non-hispanic white	Mean<br>54.3y<br>(M 53.1y<br>W 55.0y)	M&W	1) V: eating no meat, fish or poultry<br>2) SV: eating meat, fish or poultry in total < 1 time/wk<br>3) NV: eating these foods ≥1 time/wk.	FFQ	DM	1) Self-report<br>2) Physician's diagnoses	1) NV vs. V[ref]:<br>M 1.97 (1.56, 2.47)<br>W 1.93 (1.65, 2.25)<br><br>2) SV vs. V[ref]:<br>M 1.35 (1.02, 1.78)<br>W 1.08 (0.89, 1.32)	Age
7	Jaccks<br>(2016)	Cross-sectional	15,665	Centre for Cardiometabolic Risk Reduction in South-Asia (CARRS)	Urban South Asian	20-60y	M&W	1) Vegan: eating meat, poultry, fish, egg, dairy never or < 1 time/mo<br>2) LV: eating meat, poultry, fish, egg never or < 1 time/mo<br>3) LOV: eating meat, poultry, fish never or < 1 time/mo<br>4) PV: eat meat, poultry never or < 1 time/mo<br>5) SV: eat meat, poultry, fish never or ≥ 1 time/mo but < 1 time/wk<br>6) NV: no restrictions on animal-based products	Food propensity questionnaire	DM	1) Fasting blood glucose ≥126mg/dl or<br>2) HbA1c ≥ 6.5% or<br>3) Treatment of previously diagnosed diabetes with oral agents or insulin	V vs NV[ref]:<br>1.04 (0.86, 1.27)	Age, sex, education, Tabacco, alcohol, city (CARRS)
			2,159	National Health and Nutrition Examination Survey (NHANES)	US				Food propensity questionnaire			V vs NV[ref]:<br>0.75 (0.29, 1.96)	
8	Ponzio<br>(2015)	Cross-sectional	127,722	Health and Use of Health Care in Italy	Italy	Mean or range of age is not available		Not clearly defined	Questionnaire on the type of diet consumed	DM	Not available	V vs NV[ref]:<br>1.37 (1.06, 1.75)	Age, gender, education, marital status, smoking, BMI, weight control, perceived health status, hypertension, chronic disease
9	Shridhar<br>(2014)	Cross-sectional	6,555	Urban migrants, their rural siblings, urban residents of the Indian Migration Study(IMS)	India	Mean 40.9y	M&W	LV: who ate no eggs, meat, poultry, fish	FFQ	DM	1) Doctor -diagnosed and/or<br>2) Fasting plasma criterion of > 7.0 mmol/l	V vs NV[ref]:<br>0.942 (0.792-1.121)	No

10	Tonstad<br>(2009)	Cross-sectional	60,903	Adventist church members (The Adventist Health Study, AHS-2)	US, Canada: Black(black/African, West Indian/Caribbean, African, other black) nonblack(white non-Hispanic, Hispanic, Middle Eastern, Asian, Native Hawaiian/ other Pacific Islander, American Indian)	≥30y	M&W	1) Vegan: consuming no animal products (red meat, poultry, fish, eggs, milk and dairy products <1time/mo)<br>2) LOV: consuming dairy products and/or eggs(≥1 time/mo), but no fish or meat (red meat, poultry, fish <1 time/mo)<br>3) PV: consuming fish (≥1time/mo), dairy products and/or eggs but no red meat or poultry(red meat and poultry <1 time/mo)<br>4) SV: consuming dairy products and/or eggs and meat(red meat and poultry ≥1 time/mo and <1 time/wk)<br>5) NV: consuming animal products(red meat, poultry, fish, eggs, milk and dairy products >1 time/wk)	FFQ	DM	1) Fasting glucose level (≥126 mg/dl)) or<br>2) Physician-based diagnosis	1) Vegan vs. NV[ref]:<br>0.51 (0.40, 0.66)<br><br>2) LOV vs. NV[ref]:<br>0.54 (0.49, 0.60)<br><br>3) PV vs. NV[ref]:<br>0.70 (0.61, 0.80)<br><br>4) SV vs. NV[ref]:<br>0.76 (0.65, 0.90)	Age, gender, ethnicity, physical activity, education, income, TV watching, hours of sleep, alcohol consumption, BMI
11	Tonstad<br>(2013)	Prospective cohort	41,387	1) Black and non-Black participants in the Adventist Health Study(AHS)-2<br>2) Free of diabetes	US, Canada	≥30y	M&W	1) Vegan<br>2) LOV<br>3) PV<br>4) SV<br>(see above Tonstad (2009))	FFQ	DM	Self-report	1) Vegan vs. NV[ref]:<br>0.381 (0.236, 0.617)<br>2) LOV vs. NV[ref]:<br>0.618 (0.503, 0.760)<br>3) PV vs. NV[ref]:<br>0.790 (0.575, 1.086)<br>4) SV vs. NV[ref]:<br>0.486 (0.312, 0.755)	Age, BMI, gender, ethnicity, education, income, TV watching, hours of sleep, alcohol consumption, smoking, physical activity
12	Vang<br>(2008)	Prospective cohort	8,401	California Seventh-day Adventists from Adventist Mortality Study (AMS) and Adventist Health Study (AHS)	US<br>(non-hispanic white)	45-88y	M&W	1) V: consuming no meat<br>2) Individuals with occasional meat intake: consuming meat < 1time/wk<br>3) NV: consuming meat ≥1/wk	FFQ	DM	Self-report(not verified)	1) Long-term V vs NV[ref]:<br>0.746 (0.571, 0.971)<br><br>2) Long-term occasional meat intake vs. NV[ref]:<br>1.099 (0.427, 2.778)	Age, gender, education, physical activity, cigarette smoking, alcohol consumption, BMI
13	Zhang<br>(2010)	Cross-sectional	19,003<br>(M 7,148,<br>W 11,855)	Suburban residents	China	Mean 48.0y	M&W	V: consuming no meat	Questionnaire on dietary habits	DM	1) Fasting plasma glucose ≥7.0mmol/L or<br>2) Self-reported current treatment with antidiabetic medications	V vs. NV[ref]:<br>0.68 (0.55, 0.86)	Age, sex, smoking, drinking, other potential confounders

M Men, W Women, mo month, wk week, d day, LV Lacto-vegetarian, LOV Lacto-ovo-vegetarian, PV Pesco-vegetarian, SV Semi-vegetarian, NV Non-vegetarian, V vegetarian, Omni Omnivore, FFQ Food Frequency Questionnaire, DM Diabetes Mellitus, ref reference, BMI Body mass index, TG Triglycerides, LDL-C Low density lipoprotein, HDL-C High density lipoprotein, HOMA-IR Homeostasis Model Assessment-Insulin Resistence, LTPA Leisure time physical activity.
